# Supplementary material for: Inhibition of IRE1 RNase activity modulates the tumor cell secretome and enhances response to chemotherapy
Source: Nat Commun. 2018 Aug 15;9:3267. doi: 10.1038/s41467-018-05763-8 (PMC6093931; doi:10.1038/s41467-018-05763-8)
Supplement: Supplementary file 1 — Supplementary Information [file 41467_2018_5763_MOESM1_ESM.pdf]

Supplementary Information

Inhibition of IRE1 RNase activity modulates the tumor cell secretome and enhances response to chemotherapy

Logue *et al*

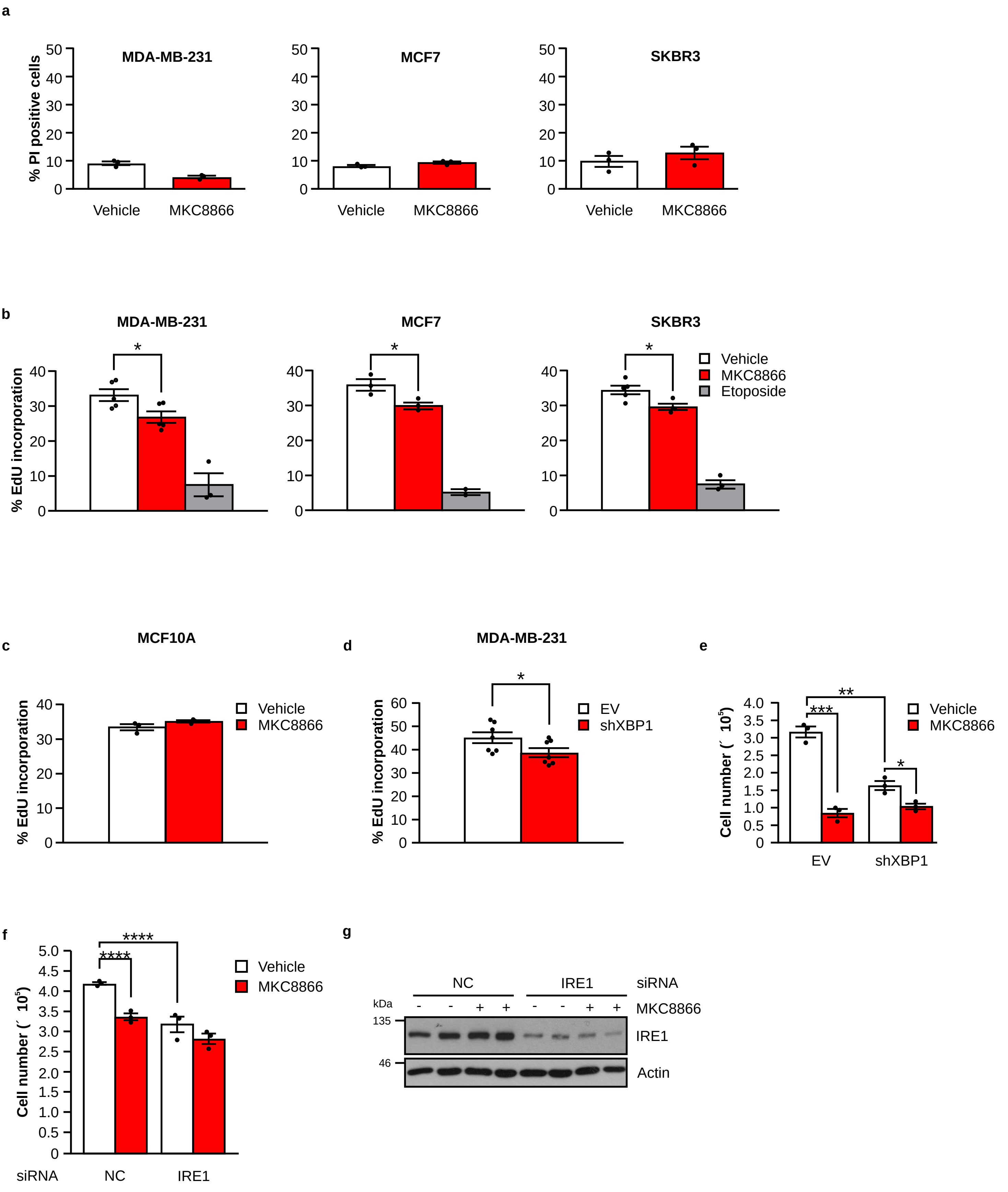

Supplementary Figure 1. MKC8866 reduces breast cancer cell proliferation. a MDA-MB-231, MCF7 and SKBR3 cells were treated with 20 mM MKC8866 or vehicle, with fresh inhibitor/vehicle added every second day. After 6 days cell death was determined by PI uptake (n = 3). **b** MDA-MB-231, MCF7 and SKBR3 cells were treated with 20 mM MKC8866 or vehicle for 48 h and the proportion of cells in S phase was assessed by EdU incorporation. To generate a positive control cells were treated for 24 h with 250 mM Etoposide (n = 3). **c** MCF10A cells treated with 20 mM MKC8866 or vehicle (n = 3), and **d** empty vector (EV) or shXBP1 MDA-MB-231 cells (n = 7) were cultured for 48 h and the proportion of cells entering S phase was assessed by EdU incorporation. **e** EV and shXBP1 MDA-MB-231 cells were seeded at equal density and treated for 5 days with vehicle alone or MKC8866 (20 mM) after which cell number was determined by cell counts (n = 3). **f**, **g** MDA-MB-231 cells were transfected with 25 nM non-targeting control (NC) or IRE1-targeting siRNA and treated as indicated with vehicle alone or MKC8866 (20 mM). After 120 h knockdown cell number was determined by cell counts, cell lysates were harvested and immunoblotted for IRE1 and Actin (n = 3 for **f**). Results shown for **e**, **f** and **g** are representative of 3 independent experiments. *P<0.05, **P<0.01, ***P<0.001 and ****P<0.0001, based on a Student's t-test. Error bars represent s.e.m.

a

GENE SIGNATURES	IRE1 HIGH vs IRE1 LOW		
	NES	FDR	P-value
SARRIO_EPITHELIAL_MESENCHYMAL_TRANSITION_UP	1.950	0.039	0
SOTIRIOU_BREAST_CANCER_GRADE_1_VS_3_UP	1.820	0.060	0.002
SMID_BREAST_CANCER_RELAPSE_IN_LUNG_UP	1.763	0.086	0.002
LANDEMAINE_LUNG_METASTASIS	1.753	0.089	0.001
ZHANG_BREAST_CANCER_PROGENITORS_UP	1.660	0.140	0.019
CHARAFE_BREAST_CANCER_LUMINAL_VS_MESENCHYMAL_UP	-2.064	0.053	0
SCHUETZ_BREAST_CANCER_DUCTAL_INVASIVE_DN	-2.044	0.035	0
POOLA_INVASIVE_BREAST_CANCER_DN	-1.939	0.083	0
SMID_BREAST_CANCER_RELAPSE_IN_BRAIN_DN	-1.919	0.084	0
SOTIRIOU_BREAST_CANCER_GRADE_1_VS_3_DN	-1.820	0.181	0

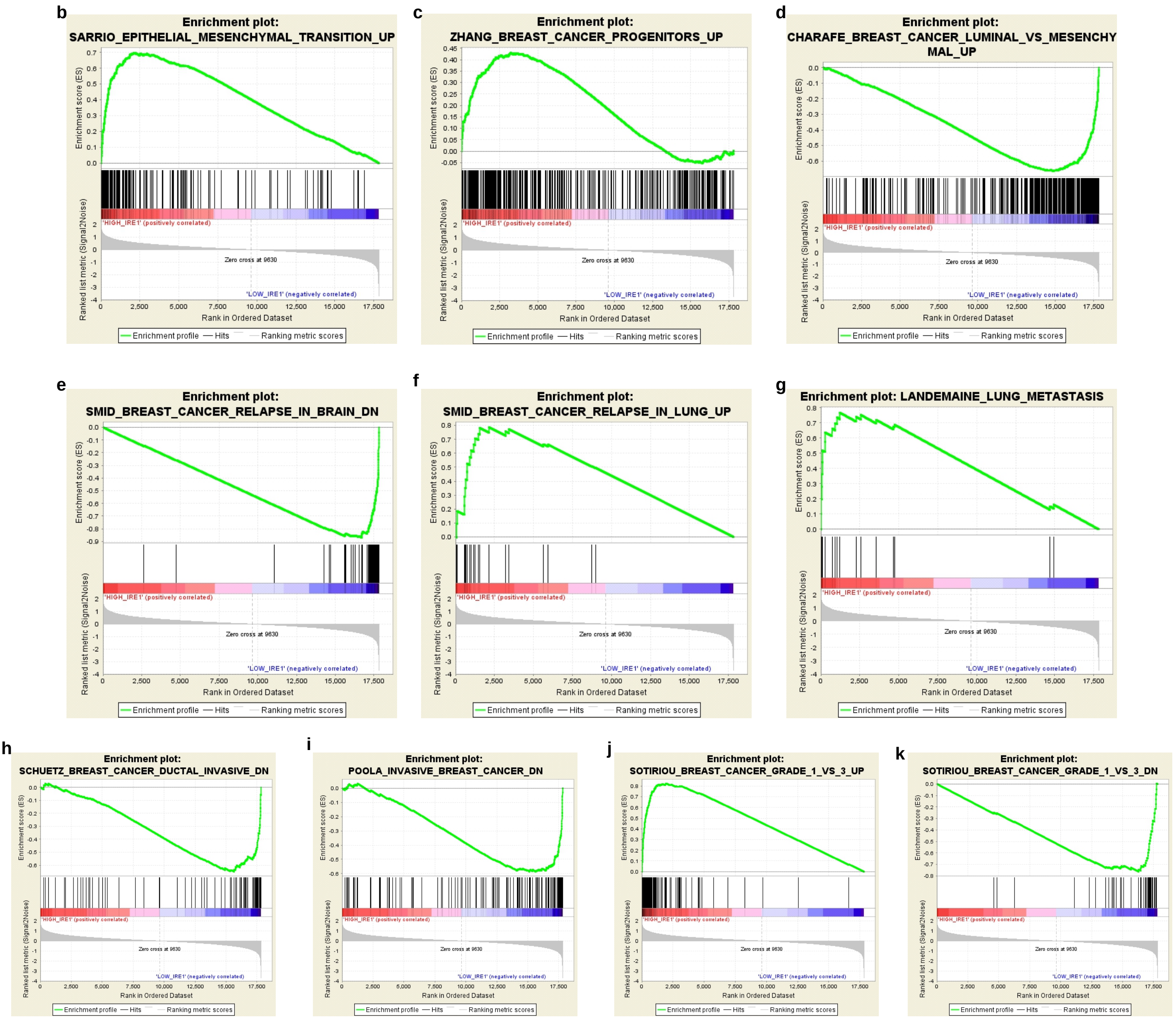

Supplementary Figure 2. High IRE1 activity associates with a mesenchymal-like phenotype and tumor progression in breast cancer data sets.
a Table illustrating the multiple breast tumor progression gene sets significantly enriched within the upregulated/downregulated genes of the high IRE1 RNase activity subgroup. NES: normalized enrichment score; FDR: false discovery rate; P-value (P-value of 0 indicates an actual P-value of less than 0.001). Gene set enrichment plots demonstrating association between the high IRE1 RNase activity group and signatures correlated with **b-d** mesenchymal-like phenotype, **e-g** tumor relapse, **h,i** tumor progression and **j,k** invasiveness.

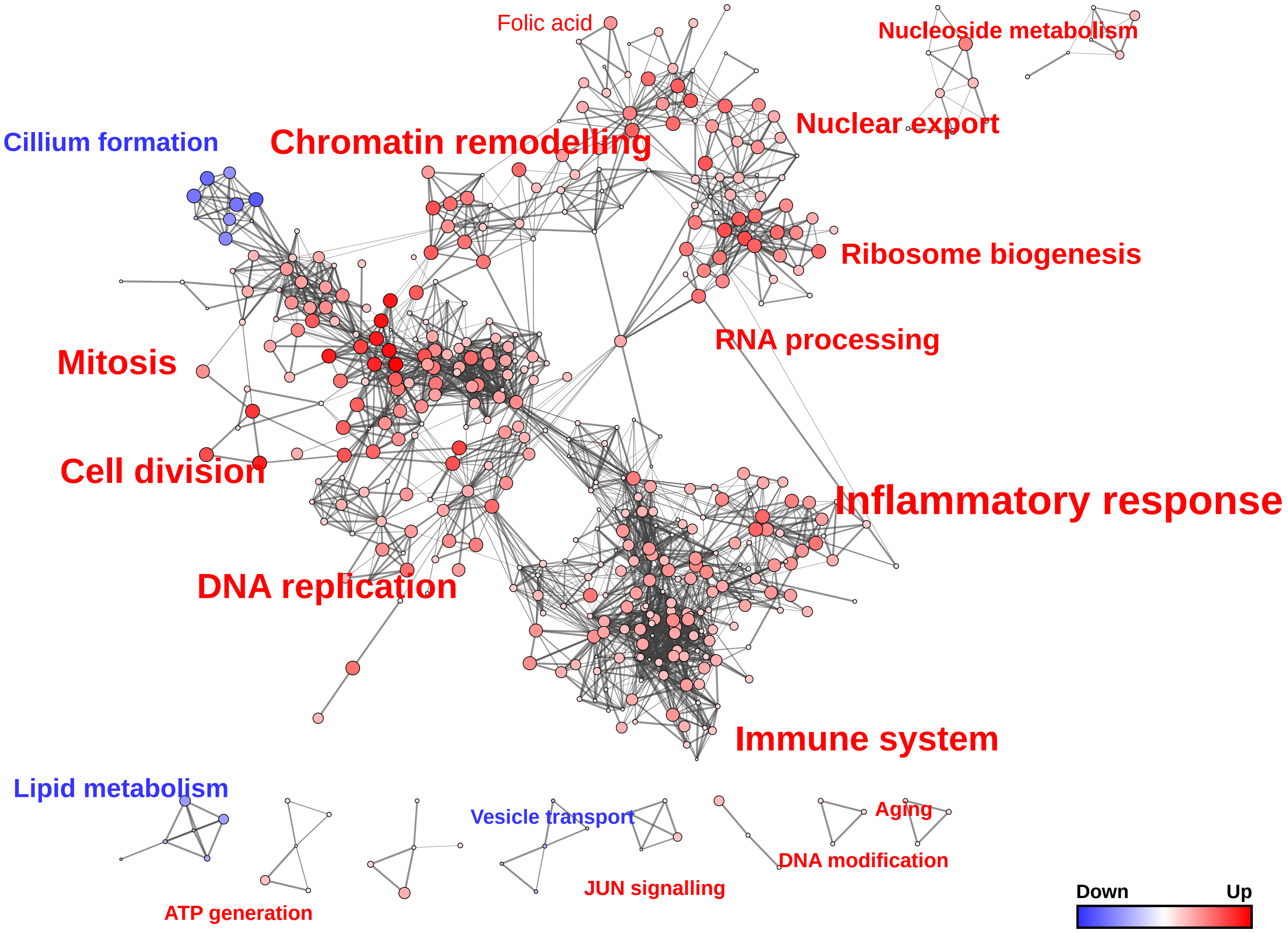

Supplementary Figure 3. IRE1 activity associates with cell proliferation, growth and immune responses. Gene ontology (GO) enriched terms among IRE1 activity correlated genes are shown. Enrichment results from GSEA are represented as a network of nodes (GO terms) linked by edges (mutual overlap between terms). Nodes are colored red to indicate positive correlation (enrichment among the positive correlated genes) or blue for negatively correlated. Annotations highlight groups of functionally related GO gene sets.

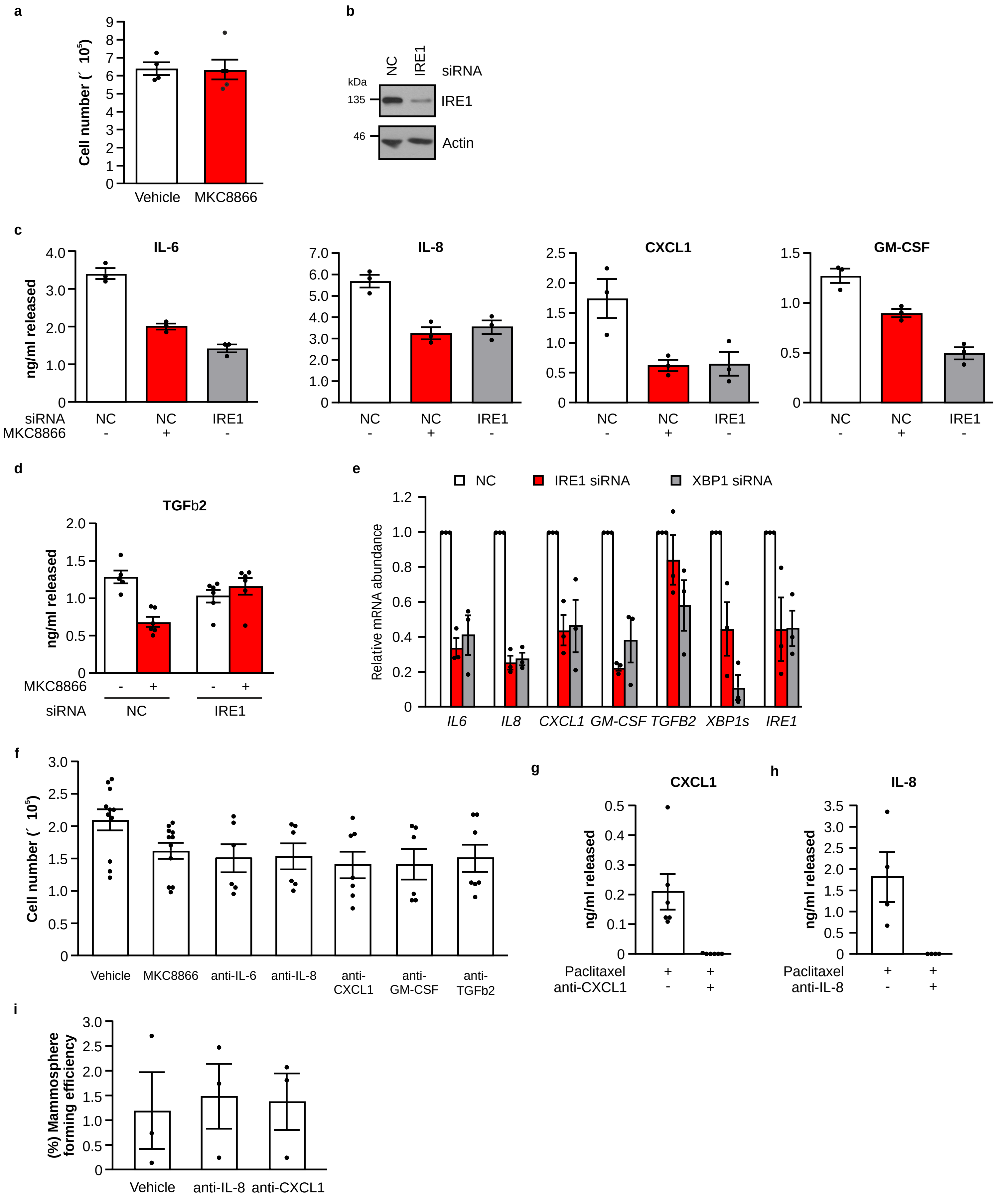

Supplementary Figure 4. Inhibition of IRE1 reduces production and secretion of pro-tumorigenic factors. a MDA-MB-231 cells where cultured in 2% serum for 48 h in the presence of 20 mM MKC8866 or vehicle after which cells were counted to confirm equal cell number (*n* = 3 cell counts). **b-e** MDA-MB-231 cells were transfected with 25 nM non-targeting control (NC) or siRNA targeting either *IRE1* or *XBP1* as indicated, and during 72 h recovery, treated with vehicle alone or MKC8866 (20 mM) as indicated. **b** Cell lysates where immunoblotted for IRE1 and Actin (representative of 3 independent experiments). **c** Conditioned medium was collected from cells and analyzed by ELISA for IL-6, IL-8, CXCL1 and GM-CSF (*n* = 3), or **d** for TGFb2 release (*n* = 5 for NC, *n* = 6 for NC/MKC8866, *n* = 6 for IRE1, *n* = 6 IRE1/MKC8866). **e** Cells were harvested and transcript levels of the indicated genes were quantified by Q-PCR (*n* = 3). **f** MDA-MB-231 cells were seeded at equal density and treated for 5 days in the presence of vehicle alone, MKC8866 (20 mM) or neutralizing antibodies against IL-6, IL-8, CXCL1, GM-CSF and TGFb2 after which cell number was determined (*n* = 6-11). **g, h** CXCL1 (*n* = 6) and IL-8 (*n* = 4) levels were analyzed by ELISA in conditioned medium from MDA-MB-231 cells treated with neutralizing antibodies against CXCL1 or IL-8 in the 72 h recovery phase post-paclitaxel treatment. **i** MDA-MB-231 cells were incubated for 3 days in the presence of vehicle alone or neutralizing antibodies against IL-8 (500 ng ml^{-1}) or CXCL1 (10 mg ml^{-1}) (*n* = 3). Cells were then counted, seeded at equal densities onto low adherence plates and mammospheres quantified after a further 5 days. Error bars represent s.e.m.

a

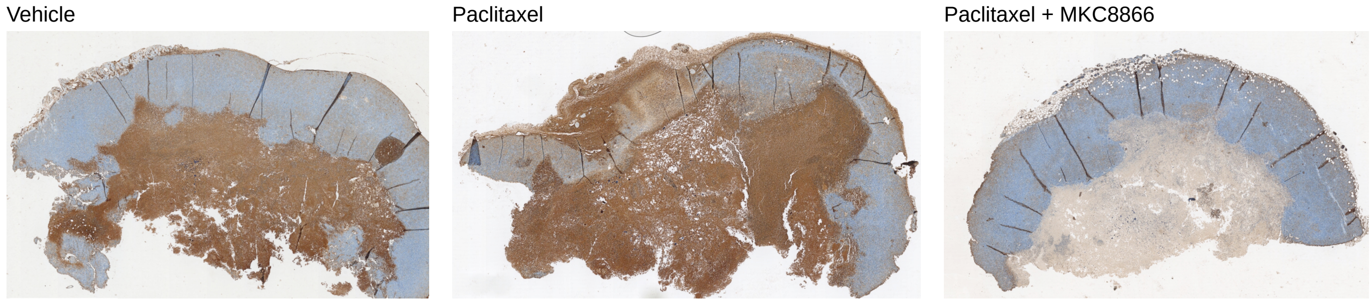

b

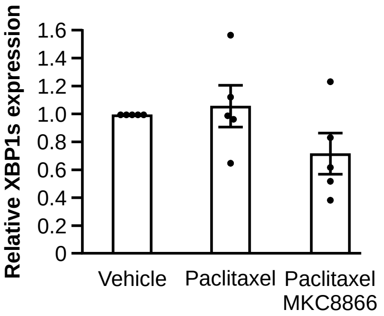

Supplementary Figure 5. Combination with MKC8866 reduces XBP1s expression in paclitaxel treated tumor xenografts. a MDA-MB-231 tumor xenografts treated with vehicle alone, paclitaxel alone or a combination of paclitaxel (10 mg kg^{-1}) and MKC8866 (150 mg kg^{-1}) were sectioned, fixed and immuno-stained using anti-XBP1s antibody. **b** Quantification of immuno-staining ($n = 5$). Error bars represent s.d.

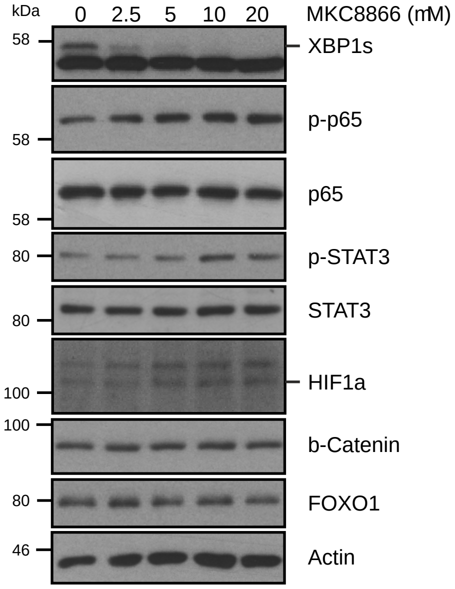

Supplementary Figure 6. Treatment with MKC8866 does not impact NF-kB, STAT3 or HIF1a activation. MDA-MB-231 cells were incubated for 72 h with vehicle alone or indicated concentrations of MKC8866 after which cell lysates were collected and immunoblotted for XBP1s, p-p65, total-p65, p-STAT3, total-STAT3, HIF1a, b-catenin or FOXO1. Actin was used as a loading control. Results shown are representative of 3 independent experiments.

Figure 1b

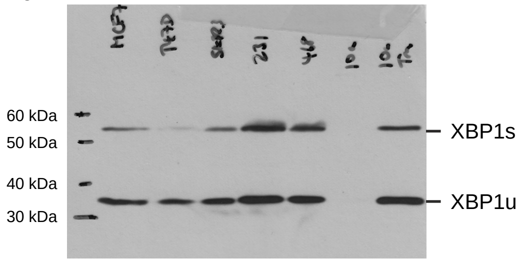

Figure 2c

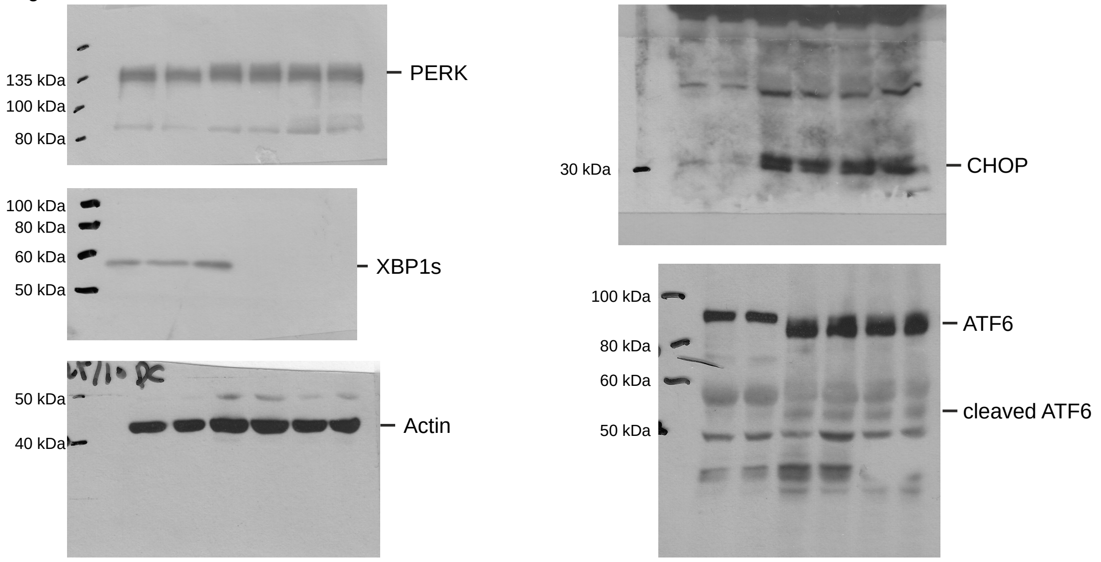

Figure 2e

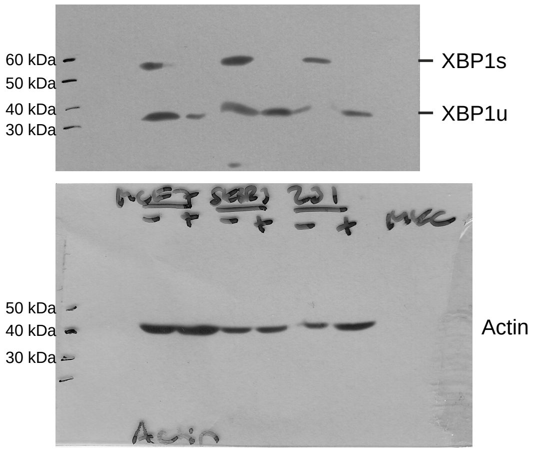

Figure 2f

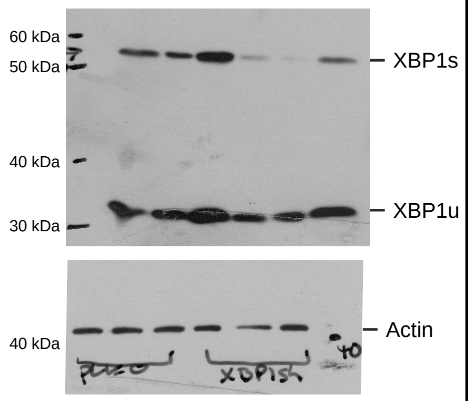

Figure 5a

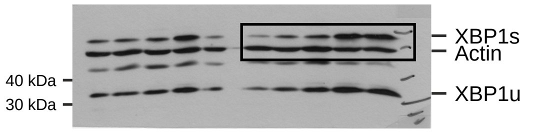

Figure 5b

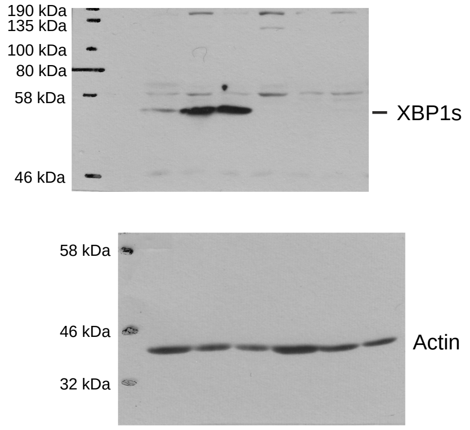

Supplementary figure 1g

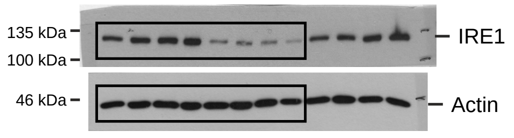

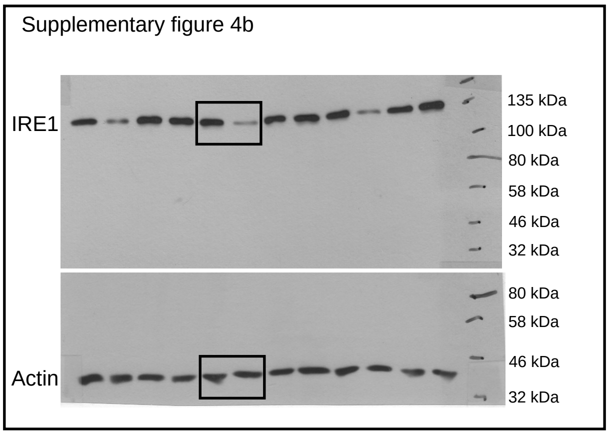

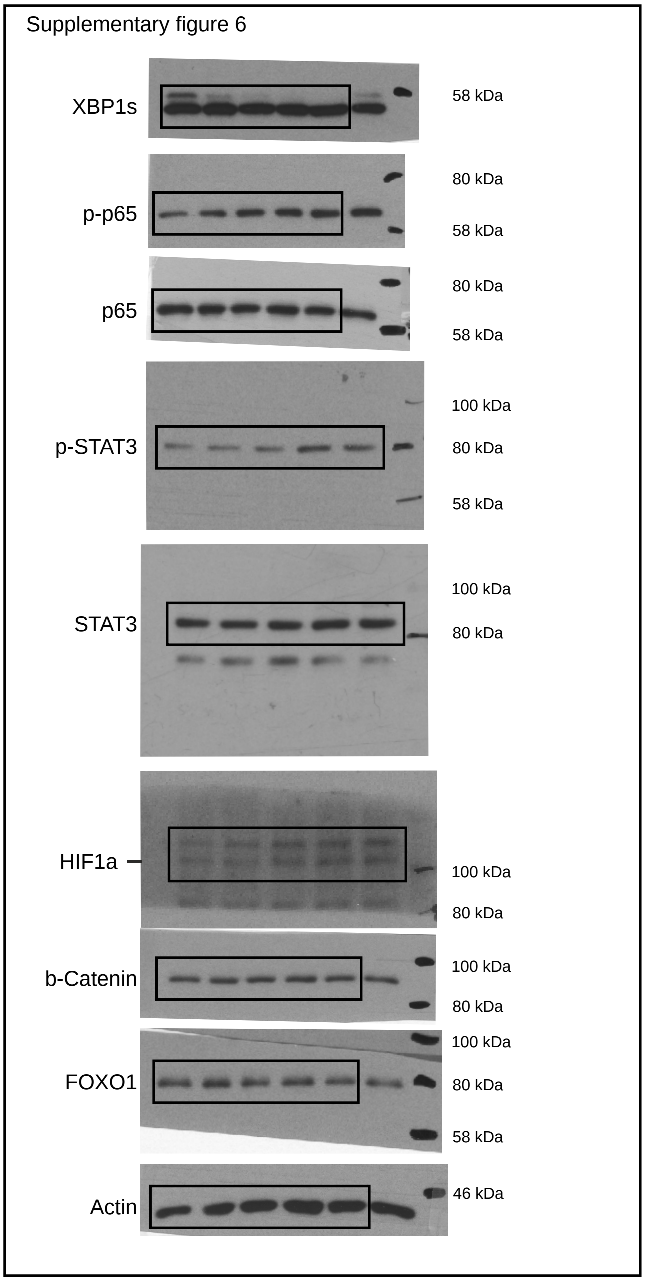

Supplementary Figure 7. Uncropped Western blots from indicated figures.

Supplementary Table 1

Composition of the IRE1 83-gene-signature generated and utilized in the study.

IRE1 gene signature (gene symbol)		
ADAT2	KIAA0020	RHOT1
ALDH6A1	KIAA0430	RIOK1
ATP7A	KRT19	RRP1
BBS2	LIPH	SERPINB2
BCAS3	LMBRD1	SLC1A1
BOP1	LTV1	SLC25A33
BYSL	MAML3	SLC5A6
C14orf1	MANSC1	SLC7A5
CCNG2	MAP3K12	SNAPC1
CYP1B1	MAP3K13	SORL1
DBP	MARS2	SPDEF
DDX21	MEGF9	SSBP2
DHCR24	MKNK2	ST6GALNAC2
DNTTIP2	MLLT3	TAF4B
DPH2	MRTO4	TCEA3
EIF5A2	MST1R	TCEAL8
FAM46C	NIP7	TEAD4
FAM63A	OCLN	TIMM8A
FDFT1	ORAI3	TINAGL1
FOSL1	PBX1	TMEM87B
FOXC1	PNO1	TOMM40
FZD7	PNPT1	TOX
GDPD1	POP1	TP53INP1
GOLPH3L	PPAT	TRERF1
GPR137B	PRICKLE2	WDR3
GTPBP4	PWP2	WDR4
HSD17B7P2	RDH11	YRDC
JUP	REPS2	

Supplementary Table 2

Combination of MKC8866 with paclitaxel enhances tumor regression compared to paclitaxel alone.

Group	N	PR	CR	TFS
Paclitaxel	10	3	1	1
Paclitaxel + MKC8866	10	8	1	0

N= Number animals in group
PR= Partial regression
CR= Complete regression
TFS= Tumor-free survival

Supplementary Table 3

Sequences of primers and probes used for PCR and Q-PCR

**PCR**		5'-3'
**GAPDH**	Fwd	ACCACAGTCCATGCCATC
	Rev	TCCACCACCCTGTTGCTG
**XBP1s**	Fwd	TCTGCTGAGTCCGCAGCAGG
	Rev	CTCTAAGACTAGAGGCTTGG
**XBP1u**	Fwd	CAGACTACGTGCGCCTCTGC
	Rev	CTTCTGGGTAGACTTCTGGG

**Q-PCR**		
**GAPDH**	Fwd	TGTAGTTGAGGTCAATGAAGGG
	Rev	ACATCGCTCAGACACCATG
	Probe	AAGGTCGGAGTCAACGGATTTGGTC
**XBP1s**	Fwd	GGAATGAAGTGAGGCCAGT
	Rev	AGAGTCAATACCGCCAGAATC
	Probe	TGAGTCCGCAGCAGGTGCA
**XBP1 Total**	Fwd	TGGATTCTGGCGGTATTGAC
	Rev	TCCTTCTGGGTAGACCTCTG
	Probe	TGGGCATTCTGGACAACTTGGACC
**PPIA**	Fwd	CATCCTAAAGCATACGGGTCC
	Rev	TCTTTCACTTTGCCAAACACC
	Probe	TGCTTGCCATCCAACCACTCAGTC
**MRPL19**	Fwd	CTTAGGAATGTTATCGAAGGACAAG
	Rev	GCTATATTCAGGAAGGGCATCT
	Probe	CTCGGGTCCAGGAGAGATTCAGGTG
**CXCL1**	Fwd	TCTCTCTTTCCTCTTCTGTTCCTA
	Rev	CATCCCCCATAGTTAAGAAAATCATC
	Probe	AAGCTCACTGGTGGCTGTTCCT
**IL6**	Fwd	GCAGATGAGTACAAAAGTCCTGA
	Rev	TTCTGTGCCTGCAGCTTC
	Probe	CAACCACAAATGCCAGCCTGCT
**IL8**	Fwd	GGGTGGAAAGGTTTGGAGTAT
	Rev	TTGGCAGCCTTCCTGATTT
	Probe	CAGCTCTGTGTGAAGGTGCAGTTT
**GMCSF**	Fwd	TGACAAGCAGAAAGTCCTTCAG
	Rev	CAGCCTCACCAAGCTCAAG
	Probe	CCAGCCACTACAAGCAGCACTG
**TGFB2**	Fwd	TGAGTCACAACAGACCAACC
	Rev	TCAATGTAAAGTGGACGTAGGC
	Probe	AAAGCAATAGGCCGCATCCAAAGC
**IRE1**	Fwd	GCATAGTCAAAGTAGGTGGCA
	Rev	GATAGTCTCTGCCCATCAACC
	Probe	TGTACGACACCAAACCCGAGAGC
